# Modulating Ocular Scarring in Glaucoma Filtration Surgery Using the Epigenetic Adjunct Suberoylanilide Hydroxamic Acid

**DOI:** 10.5005/jp-journals-10078-1246

**Published:** 2019

**Authors:** Cooper D Rodgers, Zachary L Lukowski, Jeff Min, Gina M Martorana, Mary-Kate Wilson, Jamie L Schaefer, Monica A Levine, Craig A Meyers, C Richard Blake, Gregory S Schultz, Mark B Sherwood

**Affiliations:** 1 Department of Ophthalmology, University of Florida, Gainesville, Florida, USA; Department of Ophthalmology, University of Kansas School of Medicine, Kansas City, Kansas, USA; 2 Department of Ophthalmology, University of Florida, Gainesville, Florida, USA; Department of Ophthalmology, Medical College of Georgia-Augusta University, Augusta, Georgia, USA; 3 Department of Ophthalmology, University of Florida, Gainesville, Florida, USA; Massachusetts General Hospital, Boston, Massachusetts, USA; 4 Department of Ophthalmology, University of Florida, Gainesville, Florida, USA; Department of Dermatology, University of Florida, Gainesville, Florida, USA; 5,7–9,11 Department of Ophthalmology, University of Florida, Gainesville, Florida, USA; 6 Department of Ophthalmology, West Virginia University, Morgantown, West Virginia, USA; 10 Department of Obstetrics and Gynecology, University of Florida, Gainesville, Florida, USA

**Keywords:** Epigenetics, Glaucoma, Glaucoma filtration surgery, SAHA, Suberoylanilide hydroxamic acid, Trabeculectomy, Vorinostat

## Abstract

**Aim:**

The aim of this study is to assess the effectiveness of suberoylanilide hydroxamic acid (SAHA), a histone deacetylase inhibitor (HDI) with a broad spectrum epigenetic activity, in improving filtration bleb survival as an adjunct therapy to glaucoma filtration surgery (GFS) in the rabbit model.

**Materials and methods:**

Eighteen New Zealand White rabbits underwent GFS in the left eye and were randomized to receive either a subconjunctival (SC) injection of 0.1 mL SAHA (9.25 μg/mL) or balanced saline solution (BSS) at the end of surgery, or a 3-minute intraoperative topical application of 0.4 mg/mL mitomycin-C (MMC). Bleb survival and histology were compared.

**Results:**

Blebs of rabbits receiving injections of SAHA survived an average (mean ± SD) of 23.2 ± 2.7 days. SAHA rabbits showed a nonsignificant improvement over rabbits that received an injection of BSS, which had a mean survival time of 19.7 ± 2.7 days (*p* = 0.38) according to a one-way analysis of variance (ANOVA). Eyes receiving intraoperative topical MMC survived an average of 32.5 ± 3.3 days, which is significantly longer than both the control group treated with BSS (*p* = 0.01) and the experimental group treated with the SAHA (*p* = 0.0495). SAHA was well tolerated and showed no significant avascularity, necrosis, or conjunctival thinning.

**Conclusion:**

Although it was well tolerated, a single intraoperative injection of SAHA did not significantly prolong bleb survival in the rabbit model.

**Clinical significance:**

Epigenetic adjuncts hold promise for improving GFS outcome; however, future studies must continue to examine different administration protocols and dosages to substantiate their efficacy.

**How to cite this article:**

Rodgers CD, Lukowski ZL, *et al.* Modulating Ocular Scarring in Glaucoma Filtration Surgery Using the Epigenetic Adjunct Suberoylanilide Hydroxamic Acid. J Curr Glaucoma Pract 2019;13(1):37–41.

## INTRODUCTION

When medication and laser treatment fail to reduce intraocular pressure (IOP), glaucoma filtration surgery (GFS) is the gold standard surgical procedure for patients with glaucoma.^1,2^ In GFS, aqueous humor is rerouted from the anterior chamber of the eye to the subconjunctival (SC) space, forming a filtration bleb. Unfortunately, excessive fibroblast and collagen accumulation along with increased angiogenesis often lead to scarring and subsequent filtration bleb failure.^3–5^ In humans, the antimetabolites mitomycin C (MMC) and 5-fluorouracil (5-FU) are used to help reduce the scarring and failure of glaucoma filtration blebs following GFS. However, these compounds lack specificity and can endanger the bleb's long-term structural integrity, increasing the risk of complications such as bleb leakage, hypotony, blebitis, endophthalmitis, and vision loss.^2,4,6–9^ Consequently, there is a strong interest in finding an alternative anti-scarring therapy with a lower risk of toxicity.

Epigenetics is the study of the reversible changes in gene expression which are not caused by alterations in DNA sequence. The majority of epigenetic research is focused on the effects of covalent and noncovalent modifications to DNA and histone proteins.^10^ While DNA methylation is possibly the most classic of all epigenetic modifications, other epigenetic modifications include histone methylation, acetylation, ubiquitination, and phosphorylation.^11^ Research suggests that epigenetic changes are present in a multitude of common diseases such as cancer, cardiovascular disease, and type II diabetes mellitus.^12^ Recently, evidence has emerged implicating epigenetic pathways in the pathogenesis of glaucoma.^13,14^ These pathways may downregulate neuroprotective factors important for optic nerve ganglion cell survival and intensify fibrosis in the trabecular meshwork, increasing the risk of glaucomatous progression.^15^ In addition to elucidating the underlying mechanisms behind glaucoma, epigenetic pathways provide clinicians with alternate pharmacologic targets.

SAHA (trade name Zolina; Merck & Co., Inc., Kenilworth, New Jersey) is a prototype histone deacetylase inhibitor (HDI) approved by the Food and Drug Administration (FDA) for its application in the therapy of proliferative cell conditions such as cutaneous T cell lymphoma (CTCL).^16^ SAHA has been shown to promote fibroblast apoptosis and inhibit angiogenesis, properties that would be extremely useful in filtration bleb preservation.^17,18^ The underlying mechanism behind these antifibrotic effects is not well understood, but studies suggest that HDIs may either suppress genes that promote fibrosis, such as *CTGF*, or upregulate genes that suppress fibrosis, such as *TFIGs* and *SMAD7*.^19,20^ Although studies of SAHA have shown initial positive results in corneal wound healing,^21,22^ relatively little has been done to explore SAHA's utility in glaucoma and other ocular conditions. This study evaluates the effect of a single intraoperative SC injection of SAHA on ocular scarring following GFS in the rabbit model.

## MATERIALS AND METHODS

### Study Design

Eighteen New Zealand White rabbits between 2 and 4 kg were randomized into three treatment groups (Table 1). All animal experiments were approved by the University of Florida's Institutional Animal Care and Use Committee and adhered to the ARVO Statement for the Use of Animals in Ophthalmic and Vision Research. The left eye of each rabbit underwent the GFS procedure, with the right unoperated eye serving as a control.

After randomization, rabbits in group I (*n* = 6) received a single SC injection of 0.1 mL SAHA (9.25 μg/mL) intraoperatively following the fornix-based conjunctival flap trabeculectomy. Using the same protocol, rabbits in group II (*n* = 6) received a single 0.1 mL SC injection of BSS.

Lastly, the rabbits in the positive control group (*n* = 6) were treated intraoperatively with 0.4 mg/mL MMC applied using a partial thickness Weck-cel® sponge (Alcon, Surgical, Fort Worth, TX) for 3 minutes.

### Glaucoma Filtration Surgical Procedure

The GFS procedures were performed by a single surgeon exactly as described in our previous publications.^5,23–25^ Before the procedure, a blend of xylazine (Xyla-ject, 10 mg/kg; Phoenix Pharmaceuticals, Inc.) and ketamine (Ketaject, 50 mg/kg; Phoenix Pharmaceuticals, Inc., Burlingame, CA) was injected intramuscularly for anesthesia. Proparacaine (0.1%) eye drops (Bausch and Lomb, Tampa, FL) were used as a topical anesthetic. Using a speculum, the eyelids were retracted. The eye was rotated inferonasally with corneal traction suture located in the superior quadrant. After this, the conjunctiva and Tenon's capsule were separated with blunt dissection, and a fornix-based conjunctival flap was fashioned. The rabbits in group III received a 3-minute application of 0.4 mg/mL MMC via a 4 × 4 mm^2^ Weck-cel® sponge which was placed between the Tenon's capsule and the sclera in the superior quadrant.

**Table 1:** Experimental groups

*Group*	*N*	*Treatment*
1: SAHA	6	0.1 mL SC injection of SAHA (9.25 μg/mL)
2: BSS	6	0.1 mL SC injection of BSS
3: MMC	6	Topical MMC (0.4 mg/mL) for 3 minutes

A corneal paracentesis tract was created with a #75 Beaver™ microsurgical knife (Becton Dickinson & Co., Franklin Lakes, NJ) in the superonasal quadrant and the anterior chamber was deepened by injecting viscoelastic (Healon® 10 mg/mL, Pharmacia and Upjohn) through the paracentesis. Subsequently, a 25-gauge needle was used to form a tract from the sclera to the anterior chamber. A 22-gauge angiocatheter (Insyte® Becton Dickinson Vascular Access, Sandy, UT) was then inserted along this tract. The needle was retracted, and the remaining plastic part of the cannula was carefully placed beyond pupillary margin to prevent the tip from becoming occluded by the iris. Watertight closure of the conjunctiva was achieved using an 8-0 dissolvable polygalactan (Vicryl®, Ethicon Inc., Somerville, NJ) suture at either end of the fornix-based flap and the incision was checked with fluorescein to ensure that it was Seidel negative.

At this point, rabbits in group I received a 0.1 mL SC injection of SAHA (9.25 μg/mL), and rabbits in group II received a 0.1 mL SC injection of sterile BSS. Immediately after surgery, neomycin and dexamethasone ointment was applied topically to reduce inflammation and the likelihood of infection. Rabbits in all groups received a 0.2-mL/kg oral administration of meloxicam as an analgesic for 3 days after surgery.

### Clinical Evaluations

Postoperatively, the rabbits were briefly anesthetized using isoflurane and examined every 3 days by an observer masked to the treatment type. The evaluator checked for bleb elevation and surgical complications such as corneal edema, conjunctivitis, anterior chamber shallowing, hemorrhage, bleb leakage, and lens opacification. Bleb failure was declared once the evaluator deemed the bleb flat on two consecutive occasions. The first of these occasions was recorded as the bleb endpoint.

### Histology

To compare bleb tissue from the three treatment groups at identical postoperative points while the blebs were still elevated, the eye of one rabbit from each experimental group (Table 1) was harvested at 12 days after surgery. The other eyes were only obtained after the evaluator observed that the drainage bleb was flat in two consecutive assessments. The tissue was fixed for 24 hours in a 10% neutral buffered formalin solution, imbedded in optimal cutting temperature (OCT) solution and sectioned in a sagittal plane. After preparation, the sections were stained using either Masson's trichrome or Harris hematoxylin and eosin (H&E).

### Statistical Analysis

A one-way ANOVA was used to compare the bleb survival of the three treatment groups. After this, Tukey's honest significant different (HSD) and Fisher's least significant difference (LSD) test were used to examine groups in a pairwise fashion.

## RESULTS

### Bleb Survival

**Fig. 1:**
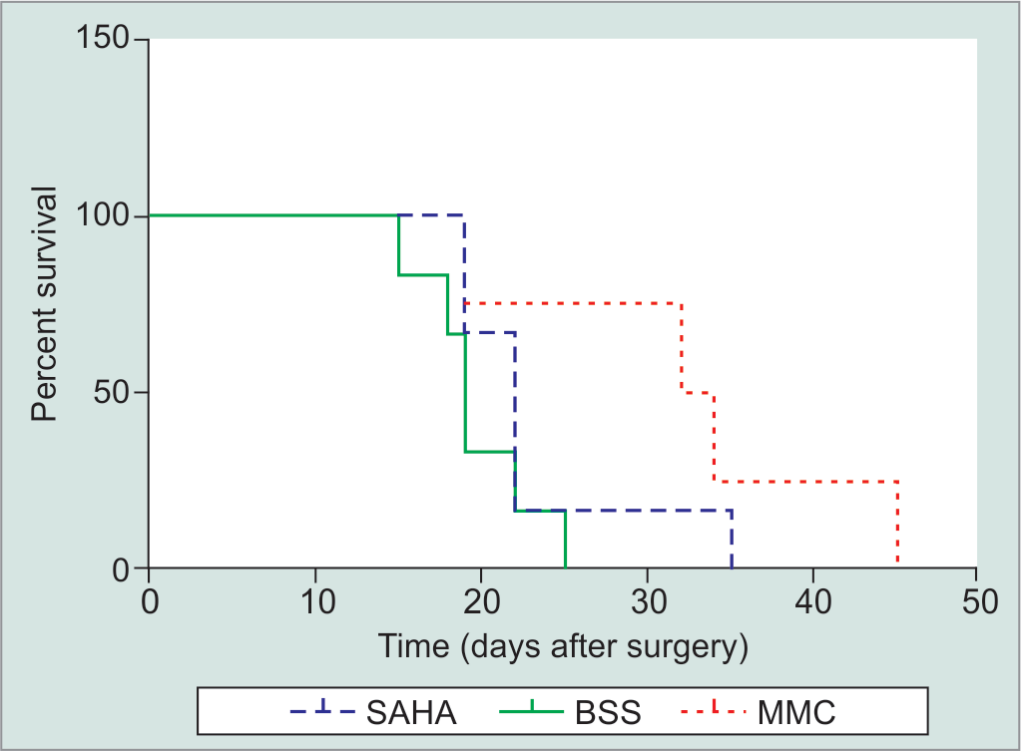
The Kaplan–Meier bleb survival plot of the eyes treated with SAHA (dashed line), MMC (dotted line), or BSS (solid line). After the masked evaluator deemed the bleb flat on two consecutive observation sessions, bleb failure was declared. The first evaluation day was recorded as the bleb endpoint

**Fig. 2:**
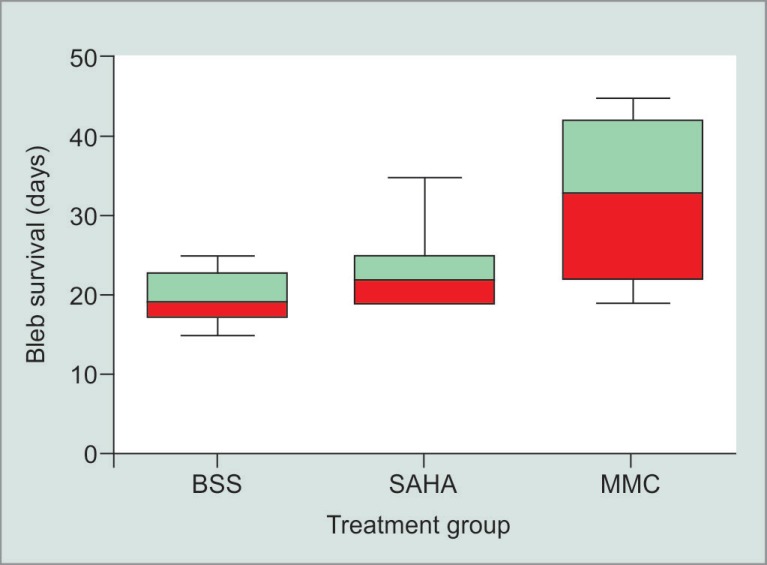
The Box-and-Whisker bleb survival plot of the eyes treated with SAHA, MMC, or BSS. After the masked evaluator deemed the bleb flat on two consecutive observation sessions, bleb failure was declared. The first of those days was recorded as the bleb endpoint

Rabbits which received an injection of SAHA had a bleb survival time (mean ± SD) of 23.2 ± 2.7 days. The average bleb survival time for the controls injected with BSS was 19.7 ± 2.7 days. The positive control group rabbits receiving MMC had an average bleb survival of 32.5 ± 3.3 days. Survival data are depicted in both Kaplan–Meier (Fig. 1) and Box-and-Whisker plots (Fig. 2).

One-way ANOVA testing showed a statistically significant difference between the three treatment groups (*p* = 0.03). *Post hoc* testing using Fisher's LSD showed that the MMC control group had significantly greater mean bleb survival than either the SAHA or BSS groups at a 95% CI (Table 2). Both Tukey's HSD and Fisher's LSD failed to show a statistically significant difference between the mean bleb survival time of rabbits treated with SAHA and rabbits treated with BSS (*p* = 0.38) at a 95% CI.

### Side Effects

SAHA was well tolerated in most of the rabbits. However, one rabbit in the SAHA experimental group was withdrawn from the study 2 days after surgery due to severe iritis. Surgical complications such as corneal edema, conjunctivitis, anterior chamber shallowing, hemorrhage, bleb leakage, and lens opacification were not present.

**Table 2:** Pairwise comparison of bleb survival

*Groups compared*	*95% Confidence interval (days)*	*p value*
SAHA vs MMC	(0.025–18.64)	0.0495
SAHA vs BSS	(−4.83–11.83)	0.380
MMC vs BSS	(3.53–22.14)	0.0107

### Histology

**Figs 3A to C:**
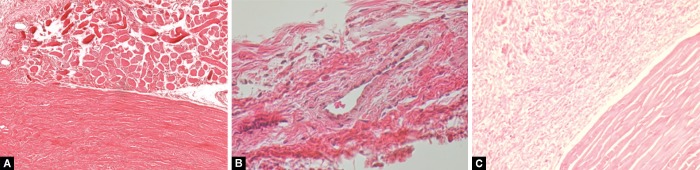
Representative sections taken 12 days after surgery stained with H&E; (A) Subconjuctival injection of SAHA; (B) Topical application of MMC; (C) SC injection of BSS

**Figs 4A to C:**
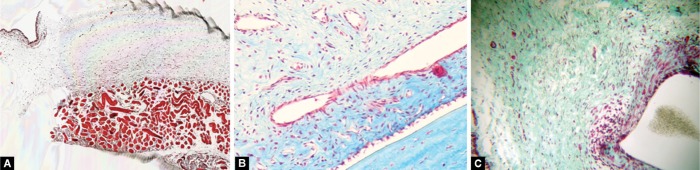
Representative sections taken 12 days after surgery stained with Masson's trichrome; (A) Subconjuctival injection of SAHA; (B) Topical application of MMC; (C) SC injection of BSS

Figures 3 and 4 are images from representative conjunctival sections from the postoperative day 12 specimens. For all experimental groups, there was an area of collagen accumulation surrounding the cannula implantation site, accompanied with fibroblast proliferation. Both the eyes receiving a SC injection of SAHA and those receiving BSS exhibited moderate collagen and fibroblast density. SAHA showed no significant acellularity or necrosis and appeared to be well tolerated.

## DISCUSSION

In comparison to the field of oncology, epigenetic therapies have been relatively unexplored in glaucoma. It is well known that fibrosis in a glaucoma bleb increases resistance to aqueous flow and contributes to the elevated IOP seen in patients with failing filtration blebs. Transforming growth factor-β2 (TGFβ2) appears to play a key role in this^26^ and is a potential target for epigenetic therapy. Bermudez et al. demonstrated that the HDI thailandepsin-A induces hyperacetylation of the TGFβ2 promoter sequence, increasing levels of TGFβ2 expression and consequent fibrosis in bovine ocular perfusion culture.^27^ This suggests that histone acetylation may play a central role in the glaucomatous change of the trabecular meshwork. Additionally, trichostatin A, another HDI, has also been shown to demonstrate promising antifibrotic effects in the rabbit model, reducing excimer laser-induced corneal haze by attenuating the TGFβ1 response.^28^

Topical SAHA has been shown to inhibit neovascularization in an alkali burn corneal injury model by attenuating hemangiogenesis, inflammatory pathways, and lymphangiogenesis.^22^ Tandon et al. investigated the efficacy of SAHA in reducing laser-induced corneal haze using both rabbit and *in vitro* models. The group found that topical application of SAHA (25 μm) for 5 minutes significantly reduced corneal haze and fibrotic marker proteins *in vivo* and TGFβ1 induced fibrosis *in vitro*, in a dose-dependent fashion without diminishing cellular viability.^21^

To date, there are only a few studies that examine SAHA and its effect on scarring in GFS. A 2008 study by Kim et al.^29^ showed that rabbit blebs treated with a 5-minute Weck-cel^®^ application of SAHA at a concentration of 300 μm survived an average of 20.3 ± 3.5 days. This was comparable to the mean survival time in our study, which was 23.2 ± 2.7 days. Also, the blebs in the Kim study MMC 0.4 mg/mL group remained elevated for the full 28 days of the study, at which time, they no longer continued to follow the blebs. Again, this is similar to the survival we observed (32.5 ± 3.3 days). Kim et al. reported that the SAHA prolonged survival in comparison to BSS. However, this group's blebs only survived a mean of 6.3 ± 1.5 days. Prior studies have shown that BSS-treated rabbit blebs typically survive much longer, for approximately 14–20 days.^30–32^ It is important to note that the researchers in this study applied SAHA topically, whereas our study delivered the SAHA through SC injection. Additionally, Kim et al. applied SAHA at approximately 8–10 times the concentration used by our group.

A 2016 study by Sharma et al.^33^ also tested the efficacy of SAHA as an adjunct to GFS. Similarly, rabbits were divided into three groups receiving a SC injection of either BSS, MMC, (0.02%) or SAHA (50 μM). The group noted an improvement in the qualitative clinical appearance of the SAHA blebs compared to the two control groups as well as a quantifiable improvement in SAHA bleb surface area compared to the BSS control. Histologically, this study showed decreased collagen deposition in the SAHA group compared to the BSS controls using H&E and Masson's trichrome. In contrast to our study, this group terminated their experiment after 14 days, choosing to focus primarily on histologic outcome measures and bleb morphometric characteristics rather than overall survival. Our group concentrated on analyzing bleb survival, which is of particular interest to clinicians.

The results of study showed that a single intraoperative SC injection of 0.1 mL SAHA (9.25 μg/mL) did not significantly prolong bleb survival over BSS. The discordance between this study and Sharma et al. may be due to differences in dosage. By mass, our group injected 0.925 μg of SAHA, while Sharma et al. injected 1.322 μg, approximately, a 43% dosage increase. Further study regarding the optimal frequency and dosing of SAHA is needed to understand the full potential of this therapy.

## CONCLUSION

Although we did not see a significant difference in bleb survival between the SAHA and BSS groups, it is possible that the dose, the method of drug delivery, and the frequency of application used in our study may not have been optimal. Further research to elucidate the most efficacious means of administering SAHA may be helpful to determine its full potential as a wound healing modulator in glaucoma surgery.

## CLINICAL SIGNIFICANCE

Although SAHA has shown promise in some previous animal model corneal ocular studies,^21,22,29,33^ our study did not confirm its efficacy as an adjunctive therapy for GFS. Additional animal studies are needed to clarify its role and the best method of application for the clinical setting in GFS.
